# Supplementary material for: Integrated Analysis of Transcriptome and Metabolome Reveals the Accumulation of Anthocyanins in Black Soybean (Glycine max L.) Seed Coats Induced by Low Nitrogen Concentration in the Nutrient Solution
Source: Plants (Basel). 2025 Sep 27;14(19):2993. doi: 10.3390/plants14192993 (PMC12526205; doi:10.3390/plants14192993)
Supplement: Supplementary file 1 [file plants-14-02993-s001.zip › Figures S1-S4.pdf]

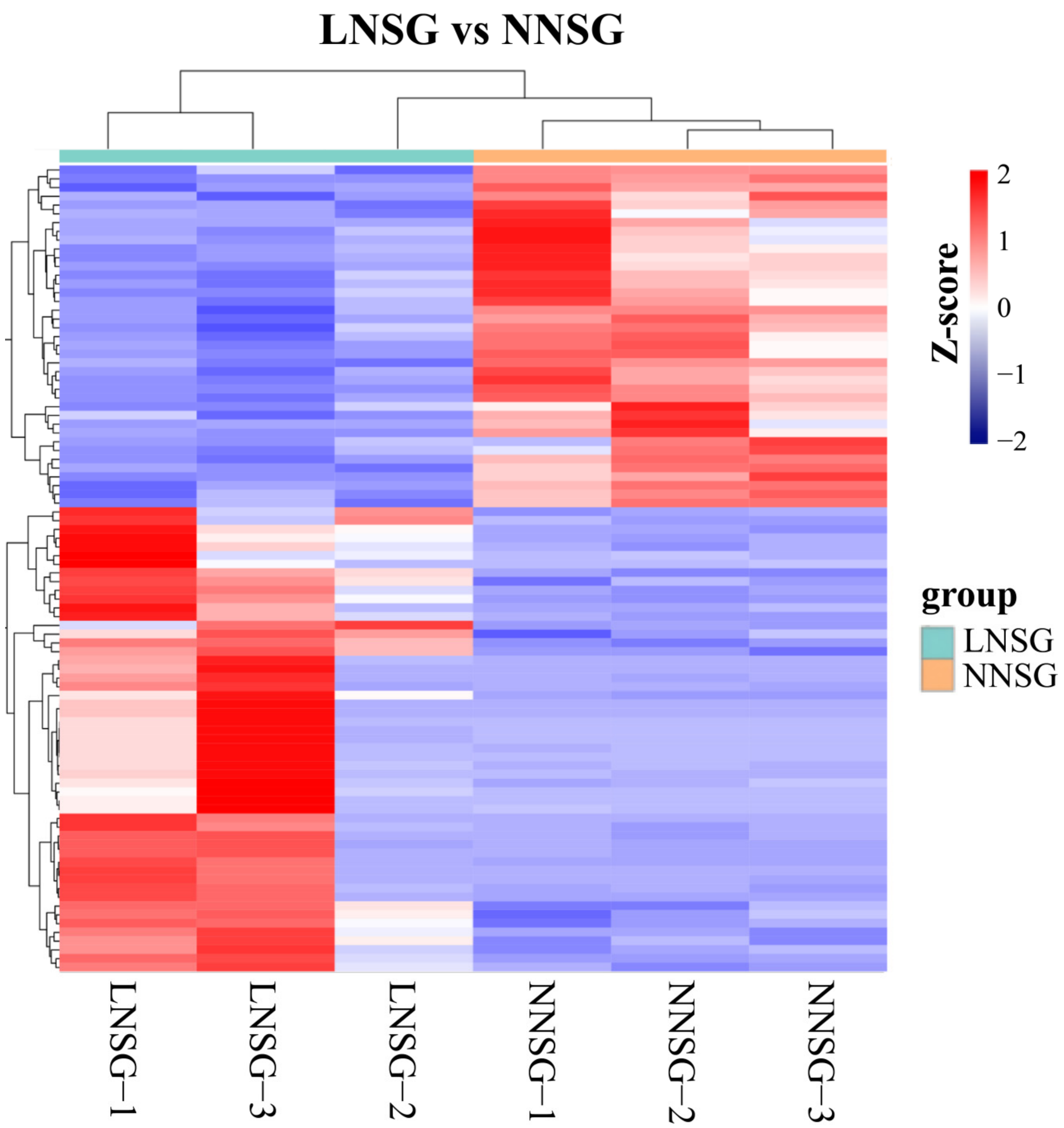

Figure S1. Cluster heatmap of differentially expressed genes between LN and NN treatment at the SG stage.

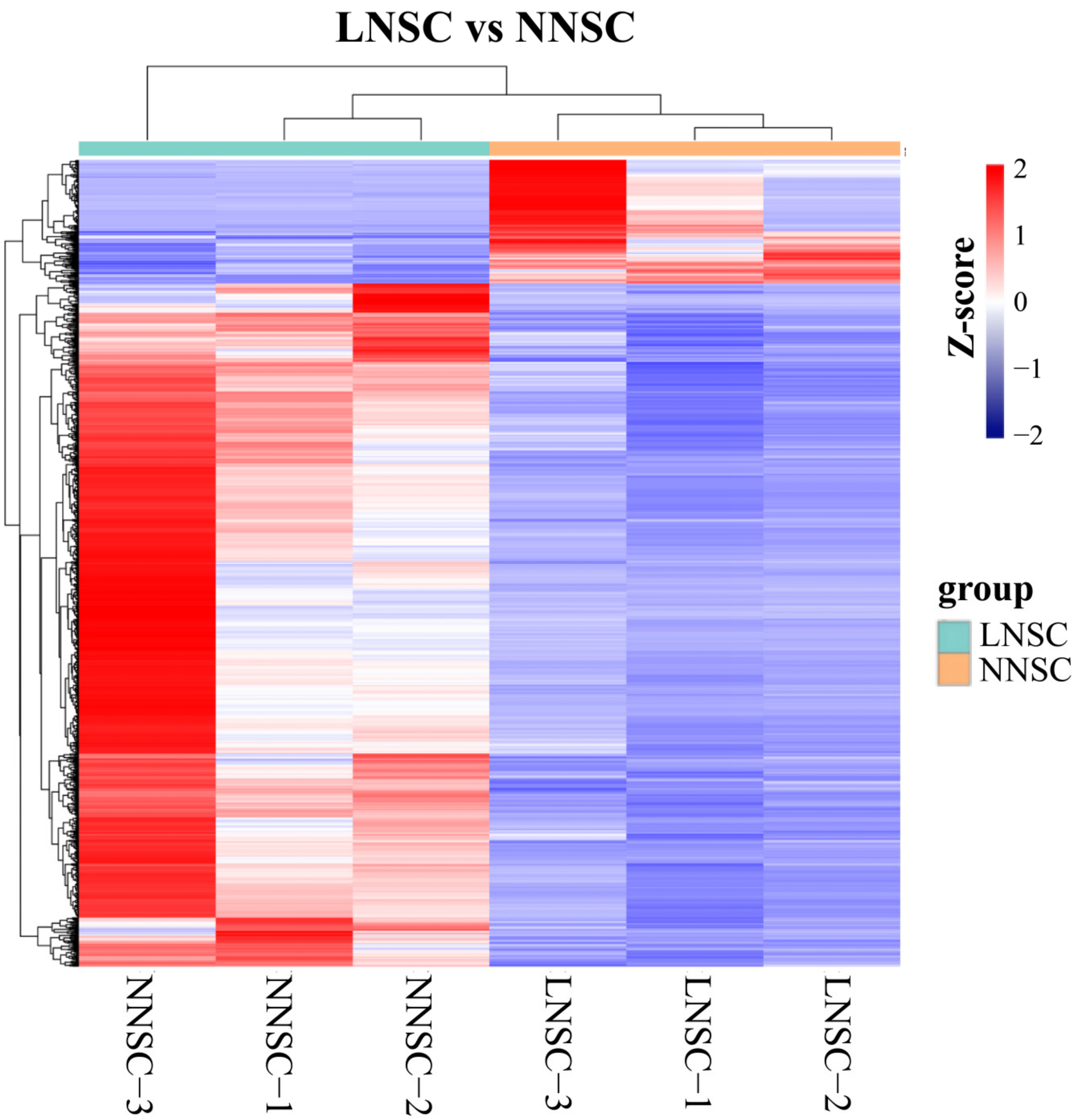

Figure S2. Cluster heatmap of differentially expressed genes between LN and NN treatment at the SC stage.

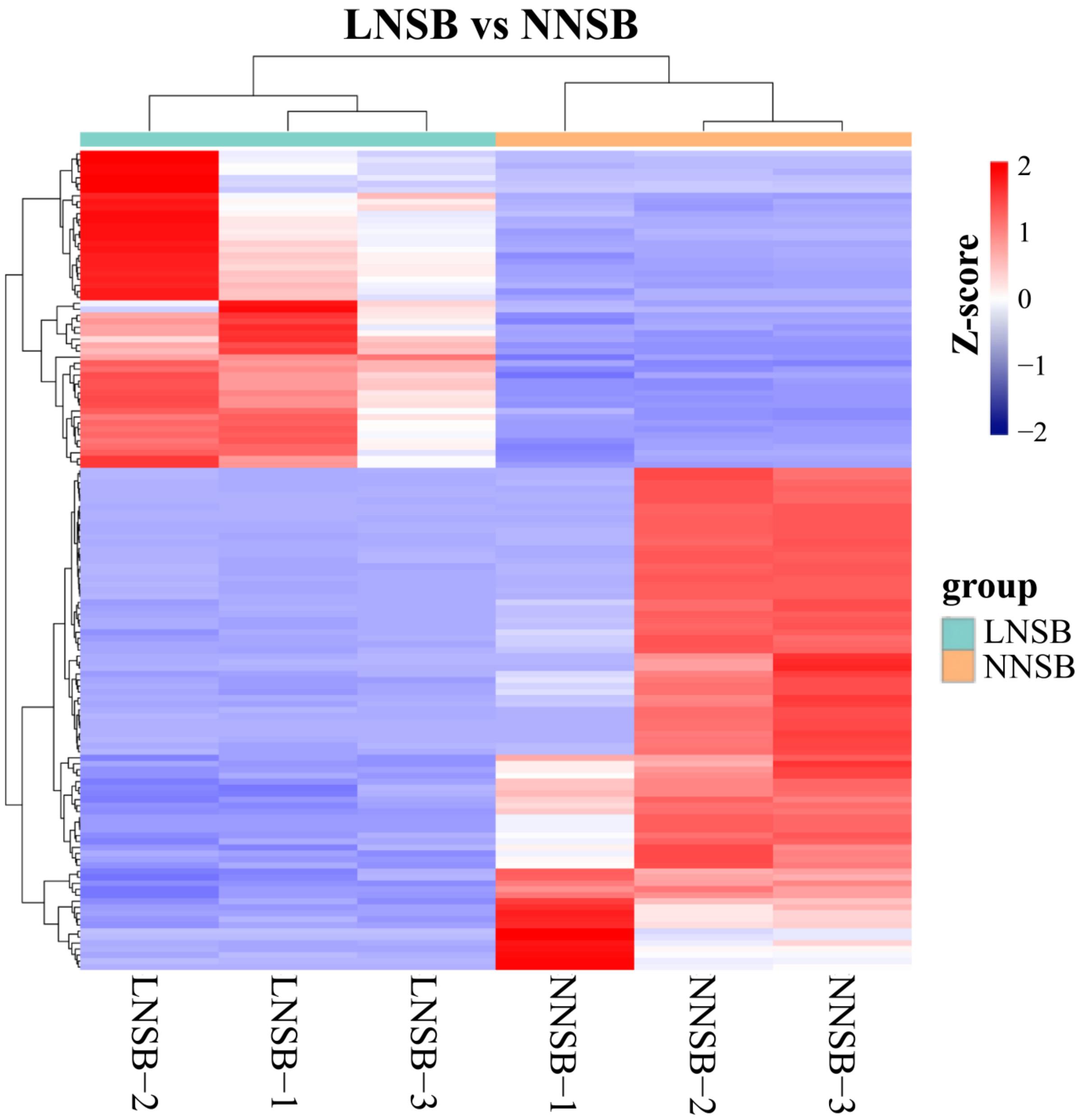

Figure S3. Cluster heatmap of differentially expressed genes between LN and NN treatment at the SB stage.

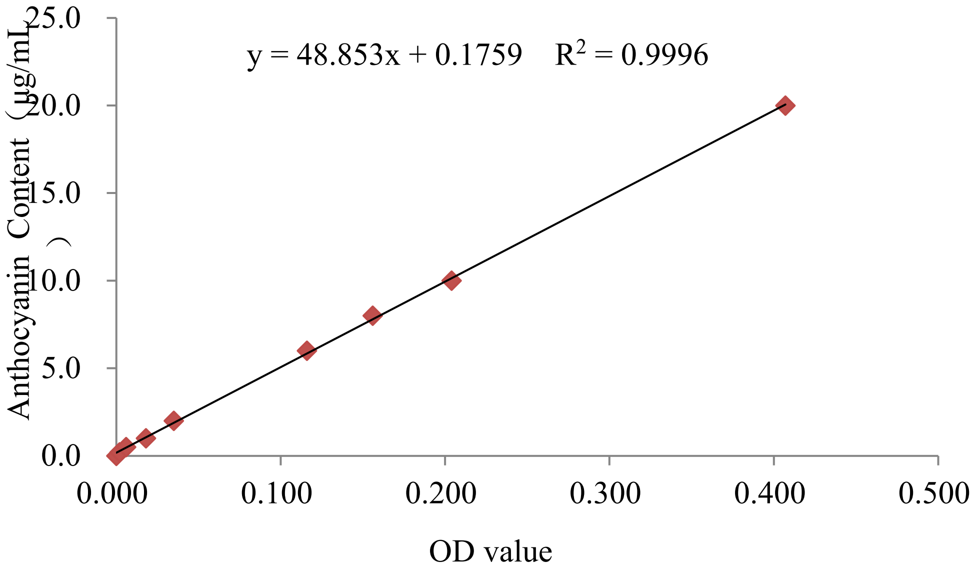

Figure S4. Standard curve of anthocyanin content
